# Effects of *Moringa oleifera* Leaf Extract on Diabetes-Induced Alterations in Paraoxonase 1 and Catalase in Rats Analyzed through Progress Kinetic and Blind Docking

**DOI:** 10.3390/antiox9090840

**Published:** 2020-09-08

**Authors:** Erick Sierra-Campos, Mónica Valdez-Solana, Claudia Avitia-Domínguez, Mara Campos-Almazán, Ismael Flores-Molina, Guadalupe García-Arenas, Alfredo Téllez-Valencia

**Affiliations:** 1 Facultad de Ciencias Químicas, Universidad Juárez del Estado de Durango Campus Gómez Palacio, Avenida Artículo 123 S/N, Fracc, Filadelfia, Gómez Palacio 35010, Mexico; valdezandival@gmail.com (M.V.-S.); loammi_greenday@hotmail.es (I.F.-M.); 2 Facultad de Medicina y Nutrición, Universidad Juárez del Estado de Durango, Avenida Universidad y Fanny Anitúa S/N, Durango 34000, Mexico; avitiaclaudia@gmail.com (C.A.-D.); marai_campos@hotmail.com (M.C.-A.); 3 Facultad de Ciencias de la Salud, Universidad Juárez del Estado de Durango Campus, Gómez Palacio 35010, Mexico; ggarrenas2000@yahoo.com.mx

**Keywords:** rat liver catalase, serum paraoxonase 1, *Moringa oleifera* leaves extract, progress kinetics and blind docking

## Abstract

In our study, we aimed to evaluate the effects of *Moringa oleifera* leaves extract on rat paraoxonase 1 (rPON1) and catalase (rCAT) activities in alloxan-induced diabetic rats. Our study included three groups; group C (control, n = 5); group D (diabetic, n = 5); and group DM (*M. oleifera* extract-supplemented diabetic rats, n = 5). Daily oral administration of *M. oleifera* extract at 200 mg/kg doses produced an increase in endogenous antioxidants. Serum rPON1 (lactonase) and liver cytosol catalase activities were determined by a spectrophotometric assay using progress curve analysis. We found a decrease in the Vm value of rPON1 in diabetic rats, but dihydrocoumarin (DHC) affinity (Km) was slightly increased. The value of Vm for the DM group was found to be reduced approximately by a factor of 3 compared with those obtained for group C, whereas Km was largely changed (96 times). Catalase activity was significantly higher in the DM group. These data suggest that the activation of rPON1 and rCAT activities by *M. oleifera* extracts may be mediated via the effect of the specific flavonoids on the enzyme structure. In addition, through molecular blind docking analysis, rPON1 was found to have two binding sites for flavonoids. In contrast, flavonoids bound at four sites in rCAT. In conclusion, the data suggest that compounds from *M. oleifera* leaves extract were able to influence the catalytic activities of both enzymes to compensate for the changes provoked by diabetes in rats.

## 1. Introduction

Diabetes is a global endemic disease with rapidly increasing prevalence in both developing and developed countries [1]. Chronic hyperglycemia generates oxidative stress in pancreatic β-cells which are particularly vulnerable to the damaging effects of producing excessive reactive oxygen species (ROS). Researchers have suggested that increased oxidative stress and changes in lipid metabolism are involved in the pathogenesis and progression of diabetic tissue damage [2,3]. A vast number of methods have been developed and used in virtually all diseases to measure the extent and nature of oxidative stress, ranging from myeloperoxidase, protein and lipid oxidation, advanced glycation end products, and their soluble receptor; none of these has reached clinical application [4]. In addition, a common practice in the assessment of tissue oxidative stress is the measurement of the maximal velocity (Vm) of antioxidant enzymes [5]. Some studies suggest that in diabetes type 2, a diminished paraoxonase 1 (PON1) activity was seen simultaneously to superoxide dismutase (SOD) decrease and a catalase (CAT) increase [6]. Moreover, there was a positive correlation between CAT/SOD, CAT/PON1 ratios and glycated hemoglobin concentration. Therefore, researchers suggest that measurement of these enzymes can be used as biomarkers of the cellular redox status. Moreover, there is an increasingly growing interest in identifying biomarkers for diseases, in which oxidative stress is involved [7].

PON1 is the best studied member of a family of enzymes called serum paraoxonases. PON1 is a calcium-dependent ester hydrolase located in a sub-fraction of high-density lipoprotein (HDL) and it is an important endogenous free radicals scavenging system in the human body [8]. An inverse relationship between PON1 activity and inflammatory responses has been described in numerous experimental models and clinical conditions, such as cardiovascular disease [9], diabetes [10], hypercholesterolemia [11], and parasite infection [12]. Therefore, low levels of PON1 have been associated with the development of several pathological conditions, whereas high levels have been shown to be anti-atherosclerotic in mouse models [13]. These findings suggest that PON1 could be a good surrogate biomarker, due to its crucial functions, inhibitors and activators of PON1 must be known for pharmacological applications. However, previous research has been shown that polymorphism account for a large degree of variation in enzyme activities in human [14]. Be this as it may, and independently of genotype, low activity of serum PON1, has been reported with oxidative stress conditions associated with uncontrolled diabetes and cardiovascular complications [15,16].

Catalase is an enzyme containing heme, and has a predominant role in protecting against the oxidative damage caused by high concentrations of hydrogen peroxide [17]. Catalase is the second most abundant enzymatic antioxidant (after SOD), which attenuates the levels of ROS that ubiquitously accompany pathological disorders such as aging, cataract, cancer, atherosclerosis, and diabetes [18]. Low catalase activity in blood is associated with the diabetes caused by alloxan [19]. However, previous studies have indicated that in diabetes and asthma, loss in catalase activity was not correlated with any decrease in its protein expression level [20,21]. Recently, it was reported that Lawsone, extracted from Henna leaves, significantly decreased Bovine liver catalase activity through a noncompetitive inhibition mechanism [22]. Additionally, in-vitro kinetics studies revealed that quercetin can inhibit catalase activity in a non-competitive manner [23]. However, ellagic acid can increase catalase activity [22]. Therefore, the effect of polyphenols on catalase activity is controversial and more studies should be carried out.

*Moringa oleifera* phytochemicals can protect liver mitochondria from oxidative injury [24]. Moreover, *M. oleifera* leaf flavonoids protect bovine mammary epithelial cells from hydrogen peroxide-induced oxidative stress in vitro [25]. In addition, *M. oleifera* leaves extract induces vasorelaxation via endothelium-dependent hyperpolarization and calcium channel blockade in mesenteric arterial beds isolated from L-NAME hypertensive rats [26]. Another study reported that *M. oleifera* extracts reversed high fructose diet-induced insulin resistance and improved testicular function [27]. Furthermore, experimental studies demonstrated that *M. oleifera* leaves extract, rich in polyphenols, delay the onset of diabetes [28,29]. Although beneficial effects of *M. oleifera* phytochemicals in reducing the risk of chronic diseases have been shown through various in-vitro/in-vivo studies [30], much more metabolic evidence is required to define a particular target(s) where the phytochemicals act as a modulator (inhibitor or activator) of diverse cellular pathways or specific enzymes. The proposed mechanisms for reducing glycemia by *M. oleifera* include inhibition of digestive enzymes and glucose uptake from the intestine, increased insulin secretion and sensitivity in diverse tissues, cytoprotective indirect antioxidant activity, and decreased gluconeogenesis in the liver [31]. However, evidence regarding changes in antioxidant enzymes due to *M. oleifera* are ambiguous because activities reported in literature vary significantly depending on the experimental conditions and remain poorly understood in animal studies [32,33]. Therefore, it is unknown whether *M. oleifera* improves the activity of antioxidant enzymes by neutralizing reactive species or affects their catalytic capacity by modifying their kinetic parameters.

Nevertheless, PON1 and CAT present complex kinetics and the results are very diverse and controversial [34,35,36]. Therefore, it is necessary to look for new methods to analyze the enzymatic activity, which could facilitate interpretation of the results.

With computer-based data-fitting methods becoming a standard tool in biochemistry, progress curve analysis of enzyme kinetics is a feasible, yet seldom used method [37]. Indeed, Golicnik and Bavec [38] reported a simple evaluation method for determining the autonomous kinetic parameter of human PON1 that could be associated with diseases. Additionally, molecular docking studies are useful to elucidate protein-ligand interactions [39]. In-silico studies have shown that phytochemicals of several plants may interact with antioxidant enzymes and pro-oxidants enzymes, explaining the therapeutic properties of these compounds [40,41].

Alloxan is known to be one of the common diabetogenic agents often used to assess the antidiabetic potential of both pure compounds and plant extracts in studies involving insulin-dependent diabetes [42]. Alloxan is a selective inhibitor of glucokinase: a glucose phosphorylating enzyme which plays a key role as glucose sensor in the pancreas and liver. In addition, alloxan is a very unstable compound, which enables it to readily undergo redox cycling. Re-oxidation of alloxan to dialuric acid causes a release of alloxan radical, which in the presence of oxygen, generates reactive oxygen species (ROS) [43]. Therefore, medicinal plant extracts with antioxidant capacity should protect enzymes against alloxan. Hence, it is necessary to have a good understanding of the kinetics parameters of PON1 and catalase to predict their diagnostic relevance as a biomarker in diabetes and to study the effects of treatment with *M. oleifera* extract. The present study aimed to comprehensively evaluate the kinetic profiles parameters of both rPON1 and rCAT and analyze the binding of phytochemicals in both enzymes, during treatment with *M. oleifera* leaves extract in a type 1 diabetes rat model. For this purpose, we carried out exploratory research to gain an in-depth understanding of how *M. oleifera* acts on antioxidant enzymes in the alloxan-diabetes model.

## 2. Materials and Methods

### 2.1. Preparation of the Extract

The extract was prepared using 23 g of dry-ground sample and 260 mL of 80% methanolic aqueous solution by successive maceration. The mixture was shaken in a magnetic grid at room temperature for 24 h and then filtered through Whatman filter paper number 1. The final extract was concentrated on a rotary evaporator, placed in a deep freezer for 24 h and lyophilized to obtain a powdered extract that was kept at −80 °C.

### 2.2. Ethics Statement

All experiments were performed in compliance with the guideline for the welfare of experimental animals by the National Institutes of Health and in accordance with the guidelines of Institutional Animal Care. This study was approved by the Institutional Animal Ethics Committee at the Faculty of Health Science, UJED with registration number (R-2019-123301538 × 0201-03).

### 2.3. Diabetic Model and Treatment

Sample size determination was performed to estimate the minimum number of animals required to significantly differentiate two groups (control and diabetic) at a *p*-value of 0.05 and confidence interval of 95% based on difference in means (glucose levels) using LaMorte’s Power Calculator [44]. A Microsoft Excel spreadsheet downloaded from the website was used [45]. A total of four animals per group was calculated, but we decided to increase this value up to five.

The Male Wistar rats were fed rat chow and water ad libitum for 30 days. Alloxan was dissolved in a citrate buffer (0.1 M, pH 4.5) and intraperitoneally injected (170 mg/kg) to induce diabetes in rats. Rats were injected only with citrate buffer served as control. Type 1 diabetes was confirmed evaluating fasting plasma glucose levels after 5 days of induction; the inclusion criteria to establish diabetes were 180–250 mg/dL of fasting plasma glucose, polydipsia, and polyphagia. Fifteen rats were used (five animals per group) that were divided in control (C group), diabetic (D group), and *M. oleifera*-treated diabetic (DM group). DM group was daily administered with a 200 mg/kg dose of extract by gavage for 3 weeks, and remaining groups were administered with distilled water as vehicle.

### 2.4. Separation of Serum

The blood was taken by cardiac puncture and allowed to clot at room temperature and serum was separated by centrifugation at 1500 rpm for 10 min and used as the source of paraoxonase 1.

### 2.5. Isolation of Liver Cytosol

The liver from rats of all groups was quickly removed after decapitation and placed in beakers containing chilled (0–4 °C) saline solution at pH 7.0. The livers were repeatedly washed with the same solution to remove blood and homogenates were prepared using a Potter-Elvehjem type glass-teflon homogenizer. The supernatant obtained after centrifugation at 12,000 rpm for 10 min was used as the source of catalase.

### 2.6. Paraoxonase 1 Activity

Lactonase hydrolytic activity of PON1 using dihydrocoumarin (DHC) as substrate (Δε270 = 1295 M^−1^ cm^−1^), was measured at 270 nm with a DR5000 Hach UV-Vis spectrometer. In an assay the cuvette contained 100 μM DHC in 50 mM Tris/HCl (pH 8, 1 mM CaCl_2_ and 1% of methanol) in a total volume of 0.6 mL. The reaction was initiated by the addition of 5 μL of plasma, and full progress curve runs were carried out in less than 5 min. The measurements for each group sample were carried in triplicate. Data were analyzed using CurveExpert professional 2.6.5 and GraphPad Prism version 8.0.1.

### 2.7. Catalase Activity

Briefly, decrease in absorbance of 5 mM solution of H_2_O_2_ in 50 mM phosphate buffer pH 7.0 for 60 s was recorded at 240 nm with a DR5000 Hach UV-Vis spectrometer. The extinction coefficient of 54 M^−1^ cm^−1^ was used for calculating activity [46]. The reaction was initiated by the addition of 1 μL (containing 28 ± 5 μg of protein) of liver cytosol, and full progress curve runs were carried out in 1 min. The measurements for each group sample were carried in triplicate.

### 2.8. Protein Determination

Protein concentration was estimated by the method of Lowry et al. [47] using bovine serum albumin, fraction V (Sigma-Meck, México) as a reference standard.

### 2.9. Kinetics Data Processing

Measurements of enzymatic reactions were used to characterize enzyme regarding their substrates affinities (Km) and maximal reactions rates (Vm). Km and Vm were determined by incubating the enzyme with varying concentration of substrate; the results usually are plotted as a graph of reaction (v) against concentration of substrate [S] yielding a hyperbolic curve. However, it is difficult to fit the best hyperbola through the experimental points, and difficult to determine Vm with any precision by estimating the limit of the hyperbola at infinite substrate concentration. Therefore, the disadvantage of this approach is that during the measurement, substrate concentrations change continuously when close to Km and also influence the rate of the reaction, as is clear from the relationship between [S] and v in Equation (1):

(d[S])/dt = v = (Vm∗[S])/(Km + [S])(1)

In addition, many measurements at different substrate concentration are needed.

Recently, Golicnik M and Bavec A [38] presented a more direct method of determining Km and Vm of paraoxonase 1 based on Lambert W function (Equation (2)):

$${\left[P\right]}_{t}={\left[S\right]}_{0}\;-\;Km*(1.45869*\ln\left(1.2*\frac{x}{\ln(2.4*\frac{x}{\ln\left(1+2.4*x\right)})}\right)-0.45869\;*\ln\left(2*\frac{x}{\ln\left(1+2*x\right)}\right)$$

where

(2) $$x=\frac{{\left[S\right]}_{0}}{Km}*\exp\left(\frac{{\left[S\right]}_{0}-Vm*t}{Km}\right)$$

However, the exact solution to the Michaelis-Menten equation, in terms of the Lambert-W function, is not available in standard curve-fitting tools and quite unfamiliar to the most researchers in the life sciences. However, modern computer software such as GraphPad Prism or Curveexpert professional, permit a mean of estimating the enzymatic kinetic parameters by this method with better accuracy and precision.

### 2.10. Rat Paraoxonase 1 and Catalase Homology Modeling

The rat paraoxonase 1 (rPON1) 3D model was built using four servers: CPHmodels −3.2 [48], swiss-model [49], Phyre2 V 2.0 [50]; and modeller 9.23 software [51]. The human PON1 crystallographic structure obtained by directed evolution (PDB ID: 1V04) was used as template [52]. The intermediate model with the best packing index according to the chosen scoring function was selected for evaluation. The stereochemical quality of each model was determined using the swiss-model server Ramachandran plot [53], and Q-Mean score [54]. The model with the best evaluation scores was selected for molecular docking studies. Finally, the two calcium ions were added using Pymol v0.99 [55]. In the case of rat catalase (rCAT) 3D model (one monomer), it was obtained following the same methodology, using as template PDB ID: 7CAT [56]. The heme group and NADPH were added into the model by superposition with the same crystal structure that served as template using Pymol v0.99.

### 2.11. Molecular Docking

The best 3D model of both enzymes was prepared adding polar hydrogens atoms and assigning Kollman charges [57] using Autodock tools [58] The structures of compounds evaluated were taken from the Pubchem database [59] and prepared for docking using the prepare_ligand4.py script from MGLTools 1.5.7 [60]. A blind docking protocol was carried out using Autodock vina v 1.1.2 [61] and, in both cases, the docking parameters were kept to their default values. The grid size was set to cover all the protein. Additionally, in both enzymes, the catalytic site was occupied, according to models built. Each compound was subjected to 10 runs with exhaustiveness value set to 8. The output file format pdbqt were converted to PDB format using OpenBabel [62]. All the images showed in the manuscript were generated using the academic version of Maestro software from Schrödinger, release 2020-1 [63].

### 2.12. Statistical Analysis

The data of three independent experiments were collected and statistically analyzed using one-way analysis of variance (one-way ANOVA), followed by Tukey’s honestly significant difference (HSD). Probability *p* < 0.05 indicated statistically significant differences.

## 3. Results and Discussion

### 3.1. Effect of M. oleifera Leaves Extract on rPON1 and rCAT Kinetics Parameters

Optimized nutrition through supplementing one´s diet with plant derived phytochemicals has attracted significant attention in preventing the onset of many chronic diseases and metabolic abnormalities, such as dyslipidemia, insulin resistance, hypertension, glucose intolerance, systemic inflammation, and oxidative stress [64]. Experimental studies demonstrate that *Moringa oleifera* leaf extracts, which are rich in polyphenols, delay the onset of diabetes [30,65]. Although various in vitro/in vivo studies demonstrate the beneficial effects of *M. oleifera* phytochemicals in reducing the risk of chronic diseases, significantly more metabolic evidence is required to define a particular target(s) where the phytochemicals act as a modulator (inhibitor or activator) of diverse cellular pathways or specific enzymes.

Enzyme kinetics constants (Km and Vm) are determined using initial velocity measurements obtained of varying substrate concentration is allowed to vary to obtain a wide range of velocities, the substrates are usually vary from 0.25 to 5 Km values [66]. A Vm value represents the maximum rate at which an enzyme can work at saturating concentrations of substrates and in the absence of products. In the cell, the flux through the enzyme may be lower than the Vm, owing to lower substrate concentrations or product inhibition. While that Km value represents the affinity of the enzyme for its substrate. Interestingly, the Michaelis-Menten constant (Km) has been suggested to be a more reliable index, compared to Vm, for monitoring structural and functional modifications of redox related enzymes [5].

PON1 has esterase and lactonase activities with a wide range of substrates, including organophosphorous compounds, non-phosphorus aryl esters and lactones, which help protect against xenobiotic toxicity. In a previous study, we reported the activity of rPON1 using the determination of the maximum velocity by the initial rates approach in the same rat model. The data showed that the values were 65.83 ± 8.9 (C group), 43.2 ± 3.5 (D group) and 107.4 ± 7.9 (DM group) nmol/min/mg protein. Compared with D group, DM group´s rPON1 activity significantly increased (2.5 times, *p* < 0.001) [67]. This agrees with the observations that PON1 activity decreases in streptozotocin-induced diabetic rats and that grape seed extract supplementation, which has a rich content of procyanidins, ameliorates PON1 activity in diabetic rats [68]. However, the kinetic parameters for various PON1 have been determined using different substrates in the presence of calcium and the Km values vary greatly [38,69]. In addition, the regulation mechanism of PON1 by flavonoids still remains unexplored. Consequently, it is difficult to make a valid comparison between the different kinetic studies.

**Figure 1:**
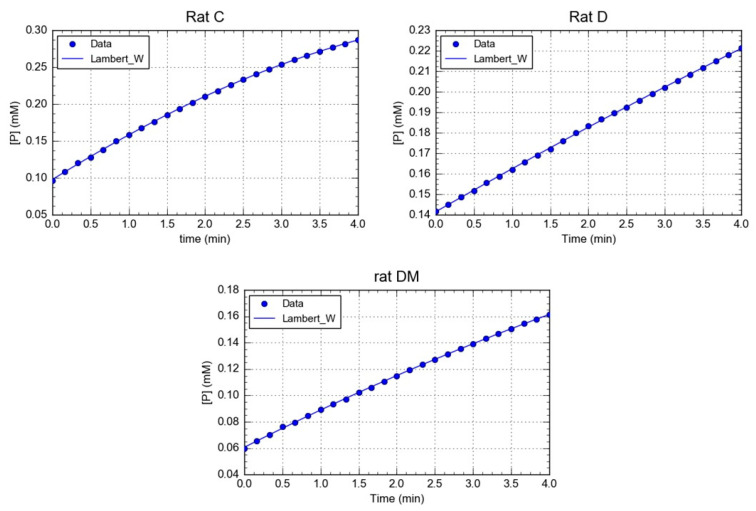
Calculation of Km and Vm from substrate concentration versus time plots. The image shows a representative trace of five repetitions for each rat of a different group.

Here, the Michaelis-Menten constant (Km) and the maximal velocity (Vm) of rPON1 in serum were determined using progress-curve kinetic analysis (Figure 1). A comparison of the kinetic data from the three groups revealed interesting results. With respect to Km values, a slight decrease was observed in the diabetic group compared with the control. However, in the DM group, the Km value diminished in nearly two orders of magnitude, demonstrating that the *M. oleifera* treatment substantially increased the affinity of the rPON1 for its substrate (Table 1). On the other hand, the Vm value varied greatly between groups C, D, and DM. In group D, the Vm was two times lower than that of the control group, whilst in the DM group, it diminished three times. Nevertheless, in general, the results kept in the same order of magnitude, and it is therefore arguable that no important changes were observed in this parameter (Table 1). However, the quotient Vm/Km drastically increased in DM compared with C and D groups, due to the *M. oleifera* treatment´s effect on Km (Table 1).

Polyphenols are known activators of PON1 [70], and it is therefore reasonable that they have a specific transcriptional role in the hepatocyte PON1 expression upregulation. Gong et al. [71] upregulated the gene expression of PON1 and suggested that quercetin has antiatherogenic properties. Furthermore, Mahrooz et al., [72] directly evaluated the effects of flavonoid naringenin on PON1 activity in human serums and purified enzymes. The arylesterase activity of purified PON1 was inhibited by naringenin up to 50% at 10 μM.

Catalase is a highly conserved antioxidant enzyme containing heme, and is known for its ability to degrade hydrogen peroxide into water and oxygen. This enzyme is the main regulator of H_2_O_2_ cellular concentration. At low concentrations, H_2_O_2_ acts a cellular messenger in insulin signaling, whereas at high concentration, it is toxic, particularly in pancreatic cells, which are catalase poor [73]. In addition, studies suggest that low catalase activity in the blood is associated with diabetes mellitus caused by alloxan administration [19]. Many phytochemicals act as agonists and increase the activity of antioxidant enzymes, including catalase [41]. Moreover, several dietary plant micronutrients that inhibit carcinogenesis, including indole-3-carbinol, indole-3-carboxaldehyde, ferulic acid, vanillic acid and epigallocatechin-3-gallate, were effective inhibitors of the oxidase activity of mammalian catalase [74]. Therefore, catalase is a potential target in many diseases, including diabetes. Drug designers should protect its catalytic function under different patho-physiological conditions [75,76] because the decrease in catalytic function would cause the accumulation of hydrogen peroxide in vivo, leading to oxidative damage in proteins and nucleic acids [77,78]. For these reasons, we used Lamber W-function to analyze of rCAT activity in our experimental groups.

**Figure 2:**
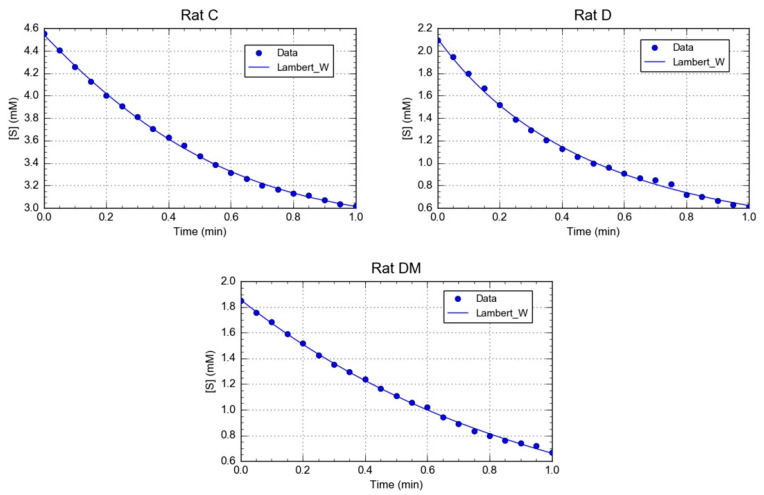
Calculation of Km and Vm from substrate concentration versus time plots. The image shows a representative trace of five repetitions for each rat of a different group.

The kinetic parameters for rCAT obtained from the progress curves (Figure 2), showed that the Km value of catalase in the control group was lower than in the diabetic and DM groups (Table 2). This result indicated that catalase from C group has a higher affinity toward hydrogen peroxide than in the other two groups. Interestingly, the *M. oleifera* leaf extracts affected the catalase binding affinity, possibly influencing the microenvironment of the catalase active site. Furthermore, the Vm value of catalase from the DM group showed a higher increase in the activity (Table 2). The results of our study agree with previous studies, showing that antioxidants such as vitamins, flavonoids and other natural compounds can increase CAT activity [79,80,81]. This result demonstrates the protective effects of *M. oleifera* on total activity; however, its impact on kinetics parameters have yet to be described.

In line with these results, abundant evidence obtained in experimental models suggests that antidiabetics offer some protection against diabetes-induced alteration in the activities of diverse antioxidant enzymes. However, its effects at the level of kinetics parameters have not been clearly elucidated. In some experiments, the primary interest is in the ratio R = Vm/Km, or more usually, Kcat/Km, which is a measurement of catalytic efficiency. This parameter is useful for comparing the effect of an inhibitor or activators on structural changes on enzyme function. In this study, the D and DM groups showed higher Vm/Km ratio values for catalase compared to the control group, due to an increase of 1.6- and 6-fold in Vm value, respectively (Table 2). At first inspection, the catalase of D group appears to be the most improved enzyme, since it displays the highest Vm/Km value. However, the H_2_O_2_ affinity of the enzyme in the D group was lower than that of the C group. Therefore, our study presents the first step in analyzing the contribution of phytochemicals of *M. oleifera* on the kinetics parameters of this enzyme in a diabetic model.

Finally, redox status expressed as CAT/SOD1 or CAT/PON1 ratios were reported as a marker for oxidative stress in diabetes. These ratios increase in diabetic patients compared to controls. Thus, these ratios may be used as markers of poor glycemic control [6]. We also calculated the CAT/PON1 ratio in each experimental group. CAT/PON1 ratio was 19.7 for C group, 63.1 for D group and 592.0 for DM group. The results revealed that alloxan affected to ratio of CAT/PON1. However, administering *M. oleifera* leaf extract to diabetic rats (DM group) significantly increased the ratio to 592.0. Therefore, the resulting CAT/PON1 ratio does not accurately reflect the redox status of cells.

Antioxidants may directly react with the reactive radicals to destroy them, or they may indirectly decreased the formation of free radicals by inhibiting the activities or expressions of ROS generating enzymes, or by enhancing the activities and expressions of other antioxidant enzymes [82]. For instance, several bioactive molecules, such as dietary polyphenols, aspirin, and its hydrolysis product salicylate, are known to stimulate PON1 transcription activation in mouse liver and HepG2 cell line [83]. In addition, S-glutathionylation or oxidized lipids may deactivate PON1 [84]. However, the regulation mechanism of PON1 by *Moringa oleifera* polyphenols remains unexplored.

On the other hand, diabetic rats treated with *Triticum aestivum* extract significantly reduced lipid peroxides, SOD and GPx and increased CAT, vitamin E and glutathione. The action of the wheatgrass demonstrates its free radical scavenging potential [85]. Thus, terrestribisamide derived from *T. aestivum* has a strong binding interaction toward catalase [41]. This suggests that the antioxidant effects of *T. aestivum* extract are exerted via modulation of catalase activity. However, the phytochemical analysis of wheatgrass showed the presence of tannins, flavonoids, saponins, and sterols, which reduce blood glucose by regenerating antioxidant systems.

In this context, diverse studies demonstrate the protective role of *M. oleifera* against oxidative stress in diverse organs of diabetic rats [86,87]. Consequently, *M. oleifera* leaves may offer a new paradigm of health care as a natural antioxidant agent due to their many beneficial health functionalities [88]. Our current investigation clearly concluded that hydroalcoholic extract of *M. oleifera* is able to control antioxidant enzymes. Furthermore, the study supports suggestions that *M. oleifera* affects rPON1 and rCAT differently, and phytochemicals of this plant act in diverse manners depending on the enzyme. Therefore, these results could be useful in developing the structure-activity relationship for serum PON1 and liver cytosol catalase activation.

### 3.2. In-Silico Identification of Potential Interactions between rPON1 and rCAT with Phytochemicals of M. oleifera

After biochemical and kinetics studies, interesting evidence emerged about how the compounds present in the methanolic extract of *M. oleifera* could be interacting with rPON1 and rCAT. Until now, no potential allosteric sites in rPON1 and rCAT has been reported to active the enzyme activity. To this end, and considering that no crystal structure is reported for either enzymes, a tridimensional model was constructed by homology modeling in each case. Four different online servers were used, as described in the methodology, and after evaluating their stereochemical quality, the best model in each case was that obtained from the program modeller (Table S1).

Structurally, the mammalian serum paraoxonase 1 (PON1) is a six-bladed β-propeller with a flexible loop (residues 71–82) covering the active site. This loop contains a functionally critical Tyr at position 71. The 3D model of rPON1 showed the same six-bladed β-propeller, with the two calcium ions located in the central tunnel. The structural calcium (Ca2) is buried, whereas the catalytic calcium (Ca1) is solvent-exposed and located at the bottom of a deep hydrophobic active site [52]. Three helices are located above the active site of rPON1, where H1 and H2 have functions in PON1-HDL interactions [89] (Figure S1).

On the other hand, the catalase (one monomer) model presented the four structural domains described in other catalases [90]. The first domain comprises of the N-terminal that, with the helix H1 and H2, forms an arm which interacts with other subunits. The second domain is integrated by helices H3-H5 and four β-sheets, giving a β-barrel folding, which is the cavity for the heme group. The third domain includes Tyr358 that is a crucial amino acid for heme group reaction. The fourth domain is formed by helices H10-H13 and is the C-terminal of the protein. Additionally, the model also contains one molecule of NADPH and the heme group (Figure S2).

According to the literature, around 120 compounds have been reported as components of *M. oleifera* leaf extracts [91,92]. In this study, after filtering the structures and taking into account the carbohydrate part, it was eliminated, and the polarity of the extract was used. Sixty compounds were selected to predict their binding mode using a blind docking protocol in both enzymes.

**Figure 3:**
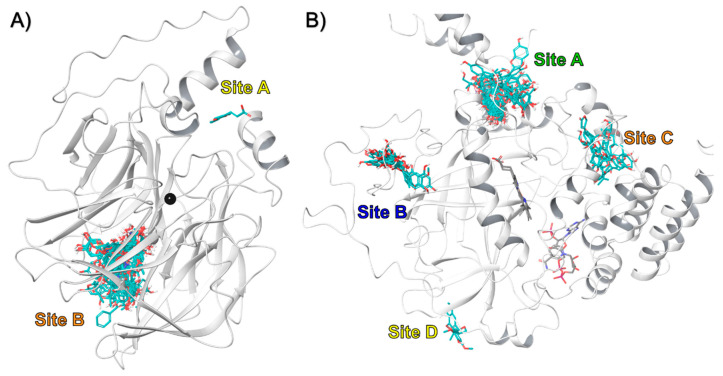
Binding of all compounds in (**A**) rPON1 and (**B**) rCAT in bind docking protocol.

**Figure 4:**
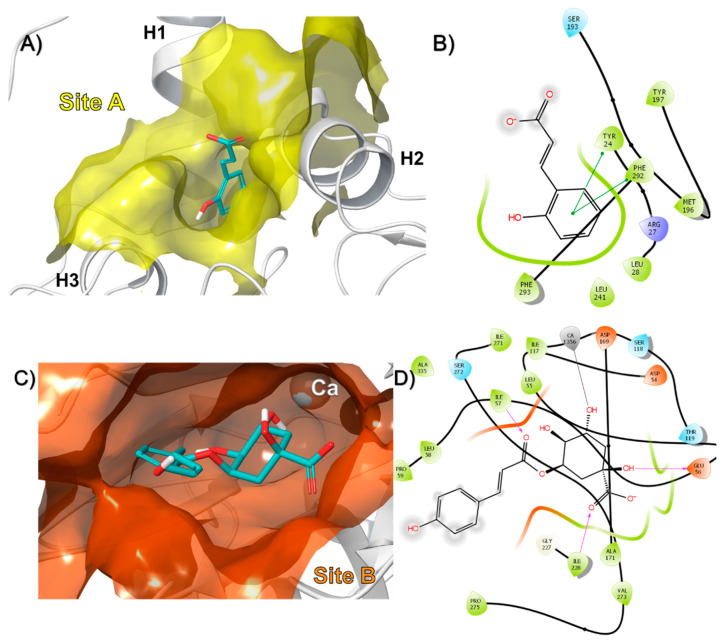
Binding mode of compound (**A**) 49 and (**C**) 26 in rPON1, as well as 2D interaction map for (**B**) 49 and (**D**) 26.

The results showed that in the case of rPON1, two binding sites were identified for these compounds, designated as A and B (Figure 3A). Interestingly, from the 60 compounds, 58 bound to the B site, and only 2 bound to the A site in the protein. For example, the o-coumaric acid (compound 49), which belongs to the group of compounds that bound in site A, formed pi-pi interactions with Tyr24 and Phe292 (Figure 4A,B). Meanwhile, that 3-p-Coumaroylquinic acid (compound 26), a representative of those that bound in site B, made hydrogen bond interactions with Glu56, Ile57, Ile228, and a metal coordination with the non-catalytic calcium ion (Figure 4C,D). The docking score of the compounds that bound to the A site of rPON1 were in the range of −5.2 to −5.9 Kcal/mol for benzyl-O-xylopyranosyl-(1→6)-β-D-glucopyranoside and o-coumaric acid, respectively. While the compounds that bound to the B site were in the range of −5.3 Kcal/mol for 4-[(α-L-rhamnosyloxy) benzyl] isothiocyanate to −11.0 Kcal/mol for mulberrofuran Q (Table S2).

Several studies in PON1 kinetics, crystallography, and mutagenesis suggest that the native PON1 substrates are lactones [93,94]. Simulation models have analyzed the type of interactions occurring between specific amino acids in the active site of the enzyme, such as Iso291, Phe292, Phe222, Asn168, Asn270, Asn224, His115 and His134, with their lactones as substrate [95]. The results from Tavori et al. also revealed an inverse correlation between docking score and rate of lactone hydrolysis, as well as a direct correlation with the length of lactone side chain. In addition, the impact of Tyr71 substitutions on PON1’s lactonase activity is minimal, whereas the kcat for the paraoxonase activity is negatively perturbed by up to 100-fold, suggesting the native activity has greater mutational robustness [89].

Our results agree with previous studies. Atrahimovic et al. [96] showed that despite the high hydrophobic subunit, the isoflavan glabridin could bind to a recombinant PON1 (re-PON1), protecting re-PON1 in a dose-dependent (1–100 μM) manner. The authors hypothesized that the mechanism governing the protective effect was not related to the antioxidant action, but rather to a physical interaction with the enzyme. The binding of glabridin affected the enzyme structure and significantly enhanced the enzyme’s ability to remove Ox-LDL associated cholesteryl ester hydroperoxides. Twelve flavonoids were docked into PON1′s potential binding site to predict the location of the flavonoid-binding site within the complete PON1 variant crystal structure, and to compare the modeling predictions with the Trp fluorescence quenching results. The approximate calculated binding energies indicated that each flavonoid bound to re-PON1 with a different affinity. The results show that the flavonoids quercetin, luteolin, apigenin, and kaempferol, which have a double bond in their C ring located in a similar site of the enzyme and superimposed each other perfectly. The interactive forces between the flavonoids and re-PON1 are mainly hydrophobic, in accordance with the Trp-fluorescence measurements. In addition, hydrogen bonds were observed between hydroxyl groups at positions 2 and 4 of the A ring and the amino acids Asp274 and Pro59 of re-PON1, respectively, and between the catechol hydroxyl groups at positions 30 and 40 of the B ring and the amino acids Asn227 and Glu56, respectively [97]. However, the re-PON1–flavonoid interaction depends not only on the number and presence of flavonoids hydroxyl groups, but also on the flavonoids substructure. In fact, although apigenin and naringenin (flavone and flavanone, respectively) have the same number of hydroxyl groups at the same positions, apigenin shows a higher affinity to re-PON1 than naringenin, likely due to a 2,4-substituted resorcinol moiety in the A ring [97].

In the case of rCAT, four binding sites were found designated as A, B, C, and D (Figure 3B). The data showed that 40 compounds bound to site A, 13 to site B, 5 to site C, and only one bound to site D in the protein. The docking score of the compounds in site A ranged from −5.1 Kcal/mol for compound 20 (benzyl-O-xylopyranosyl-(1→6)-β-D-glucopyranoside) to −10.2 Kcal/mol for compound 44 (mulberrofuran Q). For site B, the molecules with the best docking score were compounds 47 (6-hydroxykaempferol) and 48 (scutellarein) with −8.4 Kcal/mol. At site C, sophoranone was at the top with −9.3 Kcal/mol. Finally, syringaresinol mono-β-D-glucoside was the only compound that bound to site D, with a docking score of −6.9 Kcal/mol (Table S2). To our knowledge, this study reports on the molecular interaction of all these compounds with rat liver catalase for the first time.

**Figure 5:**
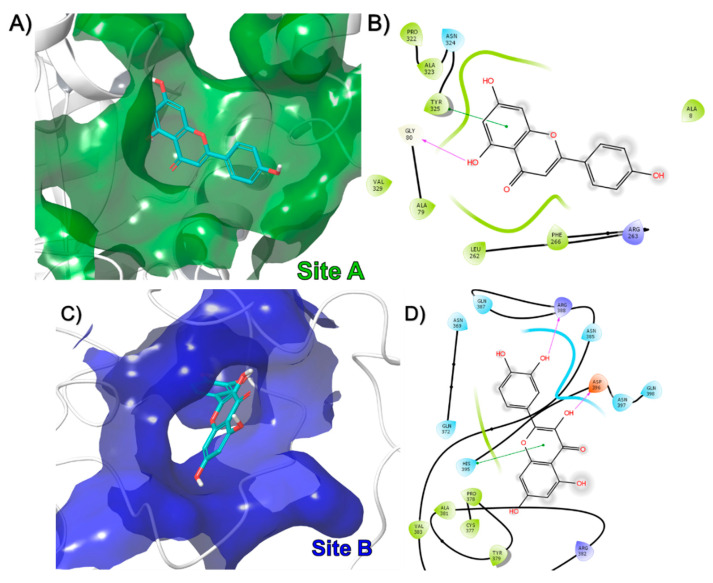

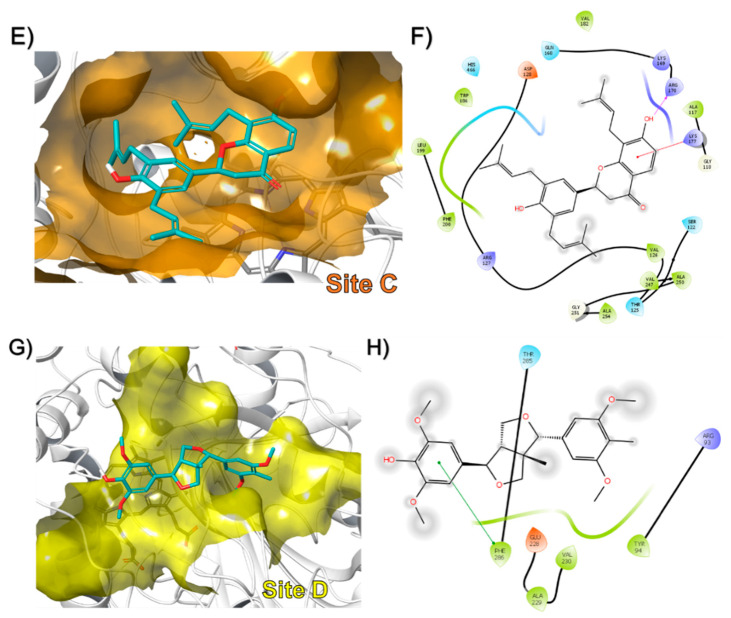
Binding mode of compound (**A**) 15, (**C**) 16, (**E**) 4, and (**G**) 43 in rat liver catalase monomer, as well as 2D interaction map for (**B**) 15, (**D**) 16, (**F**) 4, and (**H**) 43.

In a representative way, apiin (compound 15), that bound in site A, made a hydrogen bond with Gly80 and a pi-pi interaction with Tyr325 (Figure 5A,B). Quercetin (compound 16), formed hydrogen bonds interactions with Arg398 and Asp396 in site B (Figure 5C,D). Sophoranone (compound 4), made hydrogen bonds with Arg170 and a cation-pi interaction with Lys177 in site C (Figure 5E,F). Finally, syringaresinolmono-β-d-glucoside (compound 43), which was the only molecule to bind in site D, formed a pi-pi interaction with Phe286 (Figure 5G,H).

The current results were consistent with many previous studies that have been reported the interaction of phytochemical compounds such as Hesperidin and THSG [98,99,100,101,102], as well as drugs such as aspirin, levothyroxine and isoxsuprine hydrochloride with catalase [76,103]. Kinetics studies show that the bovine liver catalase increases in the presence of ellagic acid (ELA) and fluorescence analysis data reveals two binding sites for this compound on the catalase and a static type of quenching mechanism. According to molecular docking, the best binding sites for ELA are in the middle of β-barrel and wrappling domain, and in the middle of β-barrel and α-helix. Molecular dynamics simulation results show that ELA can increase catalase activity through increasing the distance between an upper side α-helix structure and a downside random coil structure [22]. Nevertheless, in-vitro kinetics studies reveal that quercetin inhibits catalase activity through non-competitive manner. Molecular docking results show that there is one binding site of quercetin on bovine liver catalase located away from heme group, at a cavity among the wrapping domain, threating arm and β-barrel. In these studies, the quercetin bound in catalase through hydrophobic and hydrogen bonds interactions, but hydrophobic were dominant [23]. Additionally, Krych and Gebicka [104] reported that various flavonoids from flavonols, flavones and catechins subclasses, efficiently reacted with catalase and inhibited its activity. Therefore, the binding sites found here are likely similar but not equivalent to that of previous studies, which suggest they can prevent its inhibition during diabetes. However, it is necessary to carry out studies with fractions enriched in each class of flavonoids of *M. oleifera* to determine the molecular interactions that mainly modulate the activity in rat liver catalase.

## 4. Conclusions

Alloxan treatment induces oxidative stress, which inhibits paraoxonase and catalase activities in rats. Indeed, the inhibition was reversed by specific changes in the paraoxonase and catalase structure by *M. oleifera* leaf extract. We found that *M. oleifera* extract stimulated higher rCAT and rPON1 values (higher Vm/Km values). The blind docking analysis showed that compounds from the methanolic leaf extracts bound in more than one site in each enzyme. These results support the view that *M. oleifera* extract inhibits the inactivation of paraoxonase and catalase by changing the affinity and catalytic capacity. Additionally, through these studies, potential druggable sites were found in paraoxonase 1 and catalase. Moreover, these data open new opportunities to study, in further detail, the role these sites play in regulating the enzyme’s activity. Although, the reported results in this study are encouraging, the study is limited by its animal model design and further clinical studies are needed to clarify whether *M. oleifera* has a similar effect on antioxidant enzymes and would facilitate comparison between animals and human studies. In addition, further structural studies are needed to determine, in more detail, the molecular interactions between flavonoids of *M. oleifera* with rPON1 and rCAT.

## Figures and Tables

**Table 1:** Kinetic parameters of rPON1 obtained from Lambert W function.

Parameters	Control (C)	Diabetic (D)	Diabetic + MOE (DM)
Km	0.4146 ± 0.04 * μM	0.325 ± 0.097 *^,†^ μM	0.0043 ± 0.0026 μM
Vm	33.15 ± 4.61 U/(min L)	17.00 ± 0.99 U/(min L) *	10.32 ± 3.36 U/(min L) *^,^^†^
Vm/Km	80.07 ± 2.8 * min^−1^	52.30 ± 9.66 * min^−1^	2400.0 ± 41.74 min^−1^

Values are mean with SD. * Statistically significant differences (*p* < 0.001) between groups were determine by one way ANOVA. ^†^ Statistically significant differences (*p* < 0.05) between groups.

**Table 2:** Kinetic parameters of rCAT obtained from Lambert W function.

Parameters	Control (C)	Diabetic (D)	Diabetic + MOE (DM)
Km	2.152 ± 0.19 mM	6.557 ± 0.42 * mM	60.7 ± 0.03 * mM
Km	652.4 ± 8 U/(min L)	1074 ± 17 U/(min L) *	6110 ± 22 U/(min L) *
Vm/Km	303.16 ± 7.8 min^−1^	1637.94 ± 15.63 min^−1^ *	1006.58 ± 10.1 min^−1^ *

Values are mean with SD. * Statistically significant differences (*p* < 0.001) between groups were determine by one way ANOVA.

## Supplementary Materials

The following are available online at https://www.mdpi.com/2076-3921/9/9/840/s1, Figure S1: The rPON1 3D model. The image shows the six-bladed β-propeller folding including the two calcium ions. The calcium ions are depicted as black and gray spheres (the black one corresponds to the catalytic calcium). Figure S2: The rCAT monomer 3D model. (A) Domains in rCAT structure. (B) The highly conserved β-barrel core structure in second domain. Table S1: Stereochemical evaluation of 3D models from rat paraoxonase 1 and catalase. Table S2: Docking score in rPON1 and rCAT obtained for each compound.
