# Supplementary material for: Effects of Moringa oleifera Leaf Extract on Diabetes-Induced Alterations in Paraoxonase 1 and Catalase in Rats Analyzed through Progress Kinetic and Blind Docking
Source: Antioxidants (Basel). 2020 Sep 8;9(9):840. doi: 10.3390/antiox9090840 (PMC7555439; doi:10.3390/antiox9090840)
Supplement: Supplementary file 1 [file antioxidants-09-00840-s001.zip › Table S1.docx]

**Table S1.** Stereochemical evaluation of 3D models from rPON1 and rCAT.

|  | **Software** | **Ramachandran plot (number of residues in)** | | | **Q-mean score** |
| --- | --- | --- | --- | --- | --- |
|  |  | **favored region** | **allowed region** | **outlier region** |  |
| Paraoxonase 1 | CPHmodels | 91.6% | 7.8% | 0.6% | -2.11 |
|  | Phyre2 | 94.8% | 4.5% | 0.6% | 0.19 |
|  | Swiss model | 94.9% | 4.8% | 0.3% | 0.19 |
|  | Modeller | 96.3% | 3.2% | 0.6% | -1.29 |
| Catalase | CPHmodels | 95.8% | 4.0% | 0.2% | -2.09 |
|  | Phyre2 | 94.3% | 5.0% | 0.8% | -2.28 |
|  | Swiss model | 96.0% | 3.2% | 0.8% | -1.18 |
|  | Modeller | 96.8% | 3.2% | 0.0% | -2.36 |
