# Supplementary material for: Effects of Moringa oleifera Leaf Extract on Diabetes-Induced Alterations in Paraoxonase 1 and Catalase in Rats Analyzed through Progress Kinetic and Blind Docking
Source: Antioxidants (Basel). 2020 Sep 8;9(9):840. doi: 10.3390/antiox9090840 (PMC7555439; doi:10.3390/antiox9090840)
Supplement: Supplementary file 1 [file antioxidants-09-00840-s001.zip › Table S2.docx]

**Table S2.** Docking score in rPON1 and rCAT obtained for each compound.

| **No.** | **Compound name and structure** | **Paraxonase-1** | | | **Catalase** | | |
| --- | --- | --- | --- | --- | --- | --- | --- |
|  |  | **Binding site** | **Docking score** | **Binding site** | | **Docking score** |  |
| 1 | Quinic acid  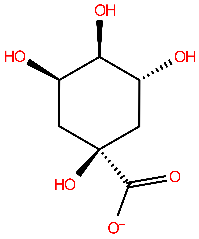 | B | -7.1 | B | | -6.1 |  |
| 2 | Cryptochlorogenic acid  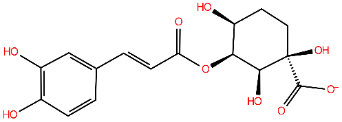 | B | -8 | A | | -7.2 |  |
| 3* | Benzoic acid 4-*O*-β-glucoside  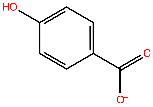 | B | -5.6 | A | | -5.5 |  |
| 4 | Sophoranone  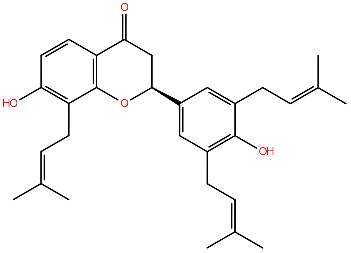 | B | -9.3 | C | | -9.3 |  |
| 5* | Marumoside  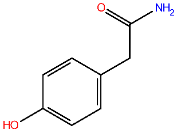 | B | -5.9 | A | | -5.8 |  |
| 6 | Neochlorogenic acid  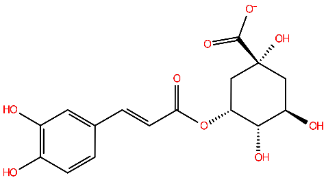 | B | -7.9 | A | | -7.4 |  |
| 7* | *O*-Ethyl-4-[(α-L-rhamnosyloxy)-benzyl]carbamate  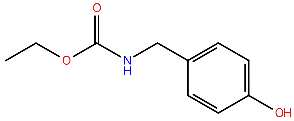 | B | -6.2 | A | | -5.2 |  |
| 8 | 3,4-Dihydroxy-benzoic acid  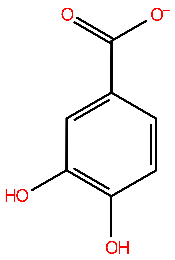 | B | -6.4 | B | | -5.7 |  |
| 9* | Benzaldehyde 4-*O*-β-glucoside  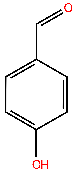 | B | -5.1 | A | | -5.2 |  |
|  |  |  |  |  | |  |  |
| **Table S2 continued…**   \| **No.** \| **Compound name and structure** \| **Paraxonase-1** \| \| **Catalase** \| \| \| --- \| --- \| --- \| --- \| --- \| --- \| \| **Binding site** \| **Docking score** \| **Binding site** \| **Docking score** \| | | | | | | | |
| 10 | Mudanpioside J  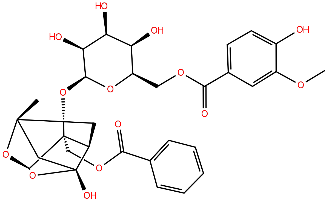 | B | -9.2 | A | | -8.7 |  |
| 11* | N, α-L-Rhamnopyranosyl vincosamide  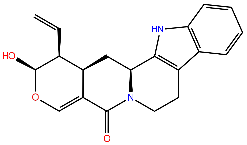 | B | -9.4 | A | | -8.8 |  |
| 12 | Dihydroquercetin  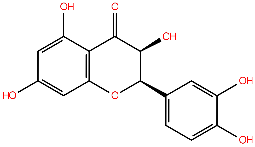 | B | -8.8 | A | | -7.5 |  |
| 13 | Chlorogenic acid  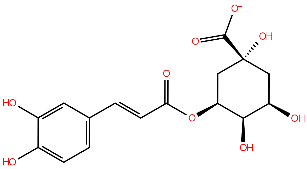 | B | -7.7 | A | | -7.5 |  |
| 14* | Tangutorid E  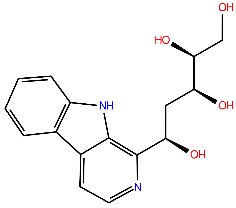 | B | -8.5 | A | | -7.3 |  |
| 15 | Apiin  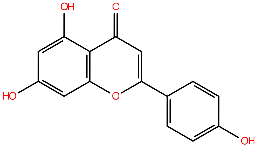 | B | -8.4 | A | | -8.1 |  |
| 16* | Quercetin-3-gentiobioside  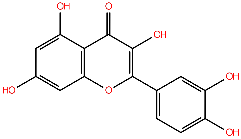 | B | -8.5 | B | | -8 |  |
| 17* | 8-*O*-Acetylshanzhiside methyl ester  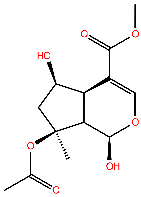 | B | -7.1 | A | | -6.3 |  |
| 18 | Caffeic acid  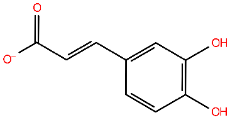 | B | -6.6 | A | | -6.3 |  |
| **Table S2 continued…** | | | | | | | |
| **No.** | **Compound name and structure** | **Paraxonase-1** | | | **Catalase** | | |
|  |  | **Binding site** | **Docking score** | **Binding site** | | **Docking score** |  |
| 19 | Methyl-4-caffeoylquinate  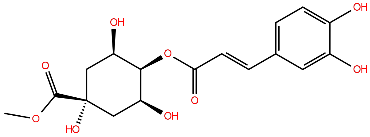 | B | -7.5 | C | | -7.2 |  |
| 20* | Benzyl-*O*-β-D-xylopyranosyl-(1🡪6)-β-D-glucopyranoside  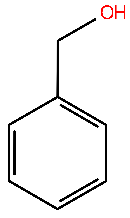 | A | -5.2 | A | | -5.1 |  |
| 21* | 6-*O*-acetylshanzhiside methyl ester  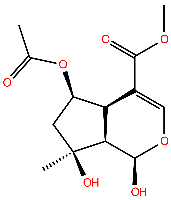 | B | -7.4 | A | | -6.5 |  |
| 22* | Niaziridin  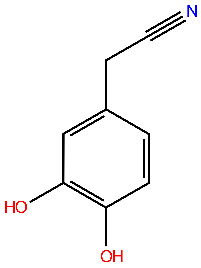 | B | -6.4 | A | | -5.8 |  |
| 23 | Vicenin-2  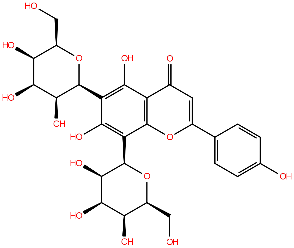 | B | -9 | C | | -7.7 |  |
| 24 | p-Coumaric acid  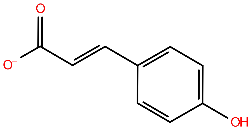 | B | -5.9 | A | | -5.9 |  |
| 25* | 6’-*O*-Benzoyl-4”-hydroxy-3”-methoxypaeoniflorin  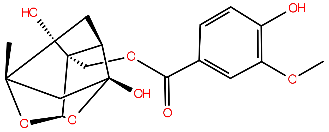 | B | -8.1 | C | | -7.3 |  |
| 26 | 3-p-Coumaroylquinic acid  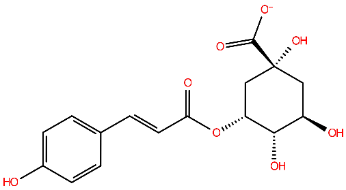 | B | -7.8 | A | | -5.9 |  |
| **Table S2 continued…**   \| **No.** \| **Compound name and structure** \| **Paraxonase-1** \| \| **Catalase** \| \| \| --- \| --- \| --- \| --- \| --- \| --- \| \| **Binding site** \| **Docking score** \| **Binding site** \| **Docking score** \| | | | | | | | |
| 27* | Kaempferol-3-*O*-rutinoside  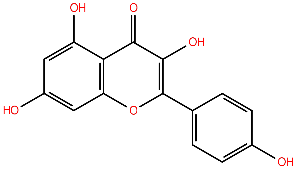 | B | -8.5 | A | | -7.5 |  |
| 28 | Padmatin  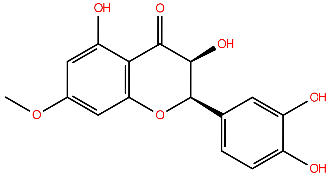 | B | -8.5 | A | | -7.6 |  |
| 29 | 4-Feruloylquinic acid  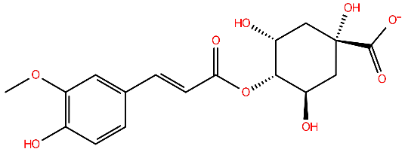 | B | -7.4 | A | | -6.9 |  |
| 30* | Niazirin  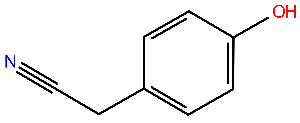 | B | -5.4 | A | | -5.6 |  |
| 31 | Epicatechin  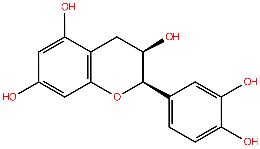 | B | -8.4 | A | | -7.6 |  |
| 32* | 3-*O*-acetyl-2-*O*-p-methoxycinnamoyl-α-L-rhamnopyranose  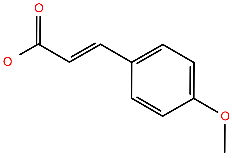 | B | -5.6 | A | | -5.6 |  |
| 33 | Astragalin  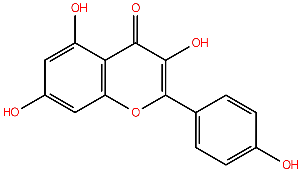 | B | -8.5 | B | | -8 |  |
| 34 | Catechin  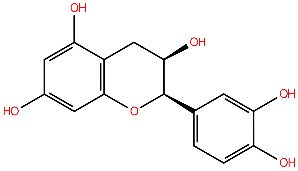 | B | -8.5 | B | | -7.5 |  |
| 35* | Apigenin-7-*O*-rutinoside  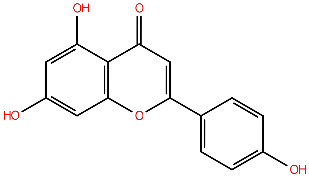 | B | -8.4 | A | | -8.1 |  |
|  |  |  |  |  | |  |  |
| **Table S2 contnued…**   \| **No.** \| **Compound name and structure** \| **Paraxonase-1** \| \| **Catalase** \| \| \| --- \| --- \| --- \| --- \| --- \| --- \| \| **Binding site** \| **Docking score** \| **Binding site** \| **Docking score** \| | | | | | | | |
| 36* | Ligan glycoside A  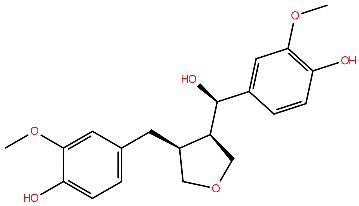 | B | -7.9 | A | | -7 |  |
| 37 | (-)-Epiafzelechin  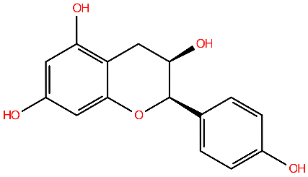 | B | -8.1 | A | | -7.4 |  |
| 38* | Isovitexin-3”-*O*-glucopyranoside  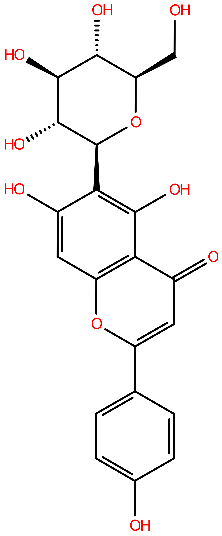 | B | -8.7 | A | | -7.5 |  |
| 39 | Procyanidins  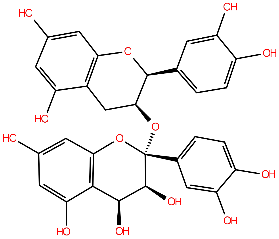 | B | -9.6 | A | | -9.1 |  |
| 40* | Isorhamnetin-3-*O*-rutinoside  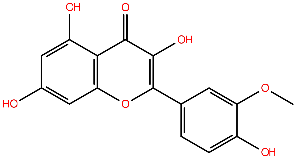 | B | -8.7 | B | | -7.6 |  |
| 41* | Chryseriol-7-O-rhamnoside  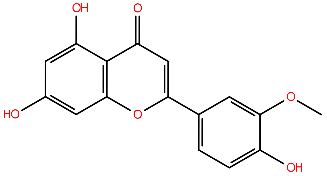 | B | -8.6 | A | | -8 |  |
| 42 | Allivictoside A  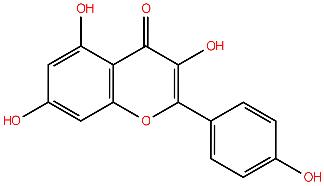 | B | -8.5 | A | | -7.5 |  |
| 43* | Syringaresinolmono-β-D-glucoside  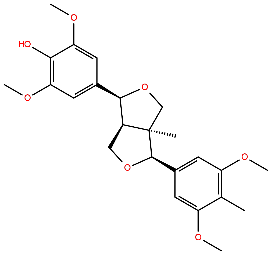 | B | -8.3 | D | | -6.9 |  |
| **Table S2 continued…**   \| **No.** \| **Compound name and structure** \| **Paraxonase-1** \| \| **Catalase** \| \| \| --- \| --- \| --- \| --- \| --- \| --- \| \| **Binding site** \| **Docking score** \| **Binding site** \| **Docking score** \| | | | | | | | |
| 44 | Mulberrofuran Q  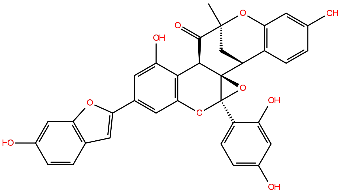 | B | -11 | A | | -10.2 |  |
| 45 | Orobol  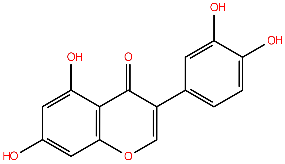 | B | -8.8 | B | | -8 |  |
| 46 | Luteolin  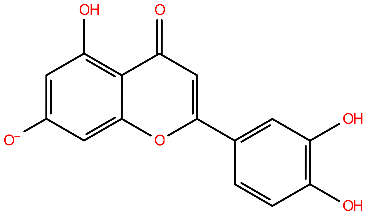 | B | -8.6 | A | | -8.2 |  |
| 47 | 6-Hydroxykaempferol  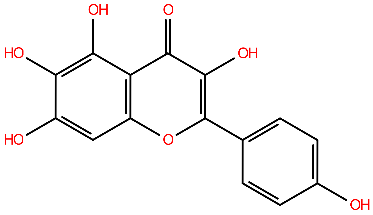 | B | -9.2 | B | | -8.4 |  |
| 48 | Scutellarein  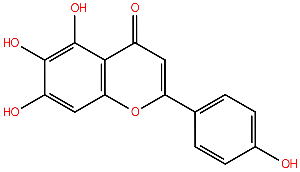 | B | -9.2 | B | | -8.4 |  |
| 49 | o-Coumaric acid  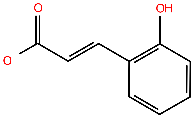 | A | -5.9 | A | | -5.9 |  |
| 50* | 7-(α-L-Galactopyranosyloxy)-5-hydroxy-2-(4-methoxyphenyl)-8-(3-methyl-2-buten-1-yl)-4-oxo-4*H*  -chromen  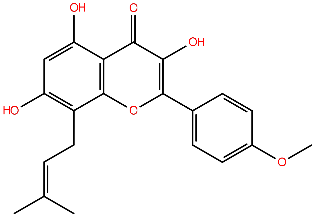 | B | -8.9 | B | | -8.2 |  |
| 51* | 4-[(α-L-rhamnosyloxy) benzyl] Isothiocyanate  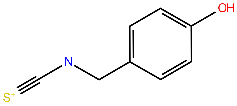 | B | -5.3 | A | | -5.4 |  |
| 52 | Rhamnetin  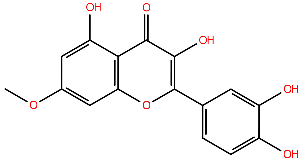 | B | -8.7 | A | | -7.4 |  |
| **Table S2 continued…**   \| **No.** \| **Compound name and structure** \| **Paraxonase-1** \| \| **Catalase** \| \| \| --- \| --- \| --- \| --- \| --- \| --- \| \| **Binding site** \| **Docking score** \| **Binding site** \| **Docking score** \| | | | | | | | |
| 53 | 5,7,20,50-Tetrahydroxyflavone  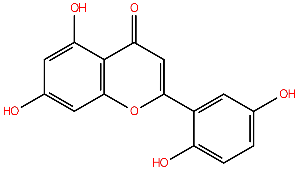 | B | -8.7 | A | | -8.3 |  |
| 54 | Eugenol  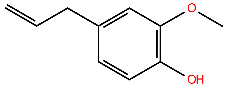 | B | -5.8 | B | | -5.8 |  |
| 55 | 1,7-Dihydroxy-2,3-methylenedioxyxanthone  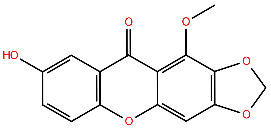 | B | -8.3 | B | | -8.3 |  |
| 56 | 2'-Hydroxygenistein  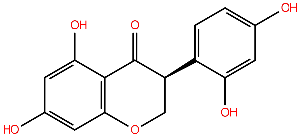 | B | -9.1 | B | | -7.3 |  |
| 57 | 3-n-Butylphthalide  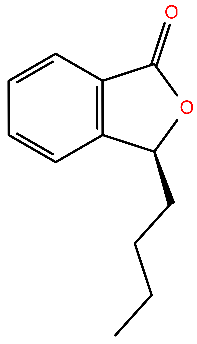 | B | -6 | A | | -6.8 |  |
| 58* | Ajugaside A  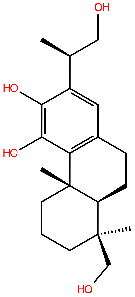 | B | -7.6 | A | | -7.6 |  |
| 59* | Diethyl phthalate  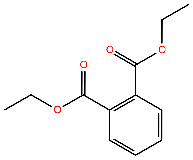 | B | -5.9 | B | | -6.1 |  |
| 60 | Erysimosole  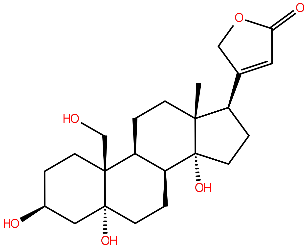 | B | -8.8 | C | | -7.4 |  |

*Compounds that were drawn without their carbohydrate part. This structure was used for blind docking studies.
