# Supplementary figures and images for: Effects of Moringa oleifera Leaf Extract on Diabetes-Induced Alterations in Paraoxonase 1 and Catalase in Rats Analyzed through Progress Kinetic and Blind Docking
Source: Antioxidants (Basel). 2020 Sep 8;9(9):840. doi: 10.3390/antiox9090840 (PMC7555439; doi:10.3390/antiox9090840)

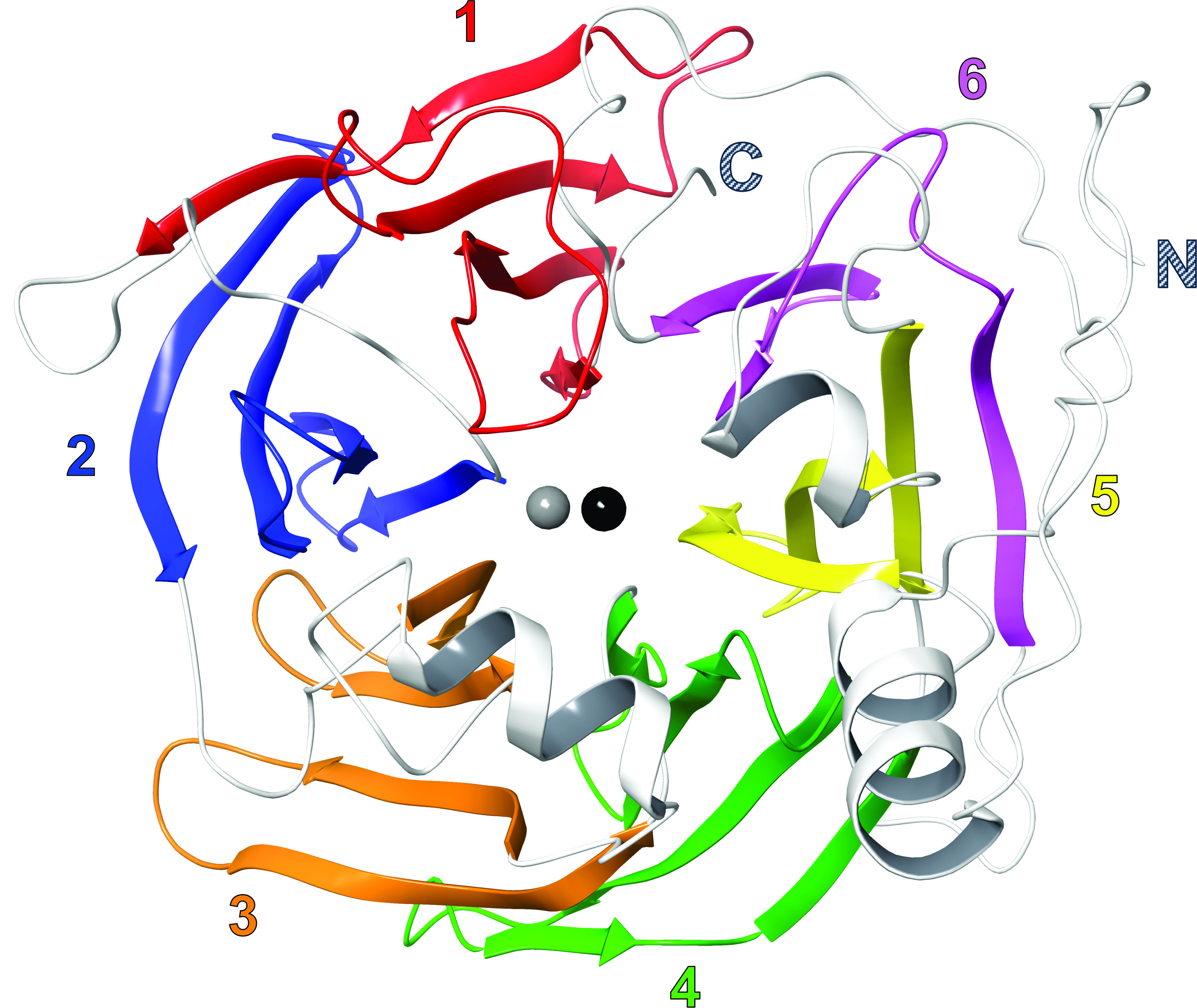

Supplement: Supplementary file 1 [file antioxidants-09-00840-s001.zip › Figure S1.jpg]

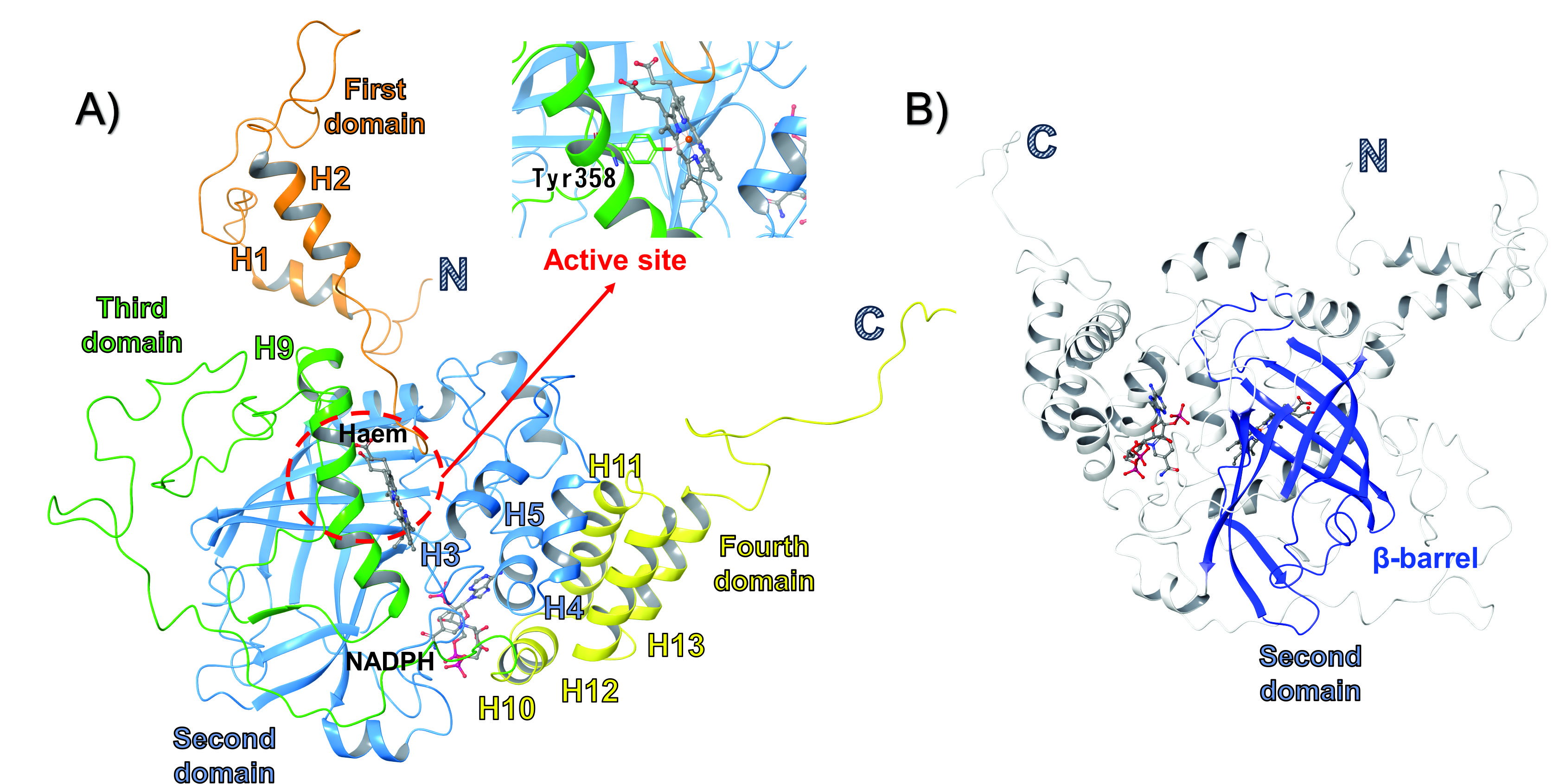

Supplement: Supplementary file 1 [file antioxidants-09-00840-s001.zip › Figure S2.jpg]
